# Folding and Biogenesis of Mitochondrial Small Tim Proteins

**DOI:** 10.3390/ijms140816685

**Published:** 2013-08-13

**Authors:** Efrain Ceh-Pavia, Michael P. Spiller, Hui Lu

**Affiliations:** Manchester Institute of Biotechnology, Faculty of Life Sciences, University of Manchester, 131 Princess Street, Manchester M1 7DN, UK; E-Mails: efrain.cehpavia@postgrad.manchester.ac.uk (E.C.-P.); michael.spiller@manchester.ac.uk (M.P.S.)

**Keywords:** oxidative protein folding, small Tim, protein import, mitochondrial intermembrane space, AAC

## Abstract

Correct and timely folding is critical to the function of all proteins. The importance of this is illustrated in the biogenesis of the mitochondrial intermembrane space (IMS) “small Tim” proteins. Biogenesis of the small Tim proteins is regulated by dedicated systems or pathways, beginning with synthesis in the cytosol and ending with assembly of individually folded proteins into functional complexes in the mitochondrial IMS. The process is mostly centered on regulating the redox states of the conserved cysteine residues: oxidative folding is crucial for protein function in the IMS, but oxidized (disulfide bonded) proteins cannot be imported into mitochondria. How the redox-sensitive small Tim precursor proteins are maintained in a reduced, import-competent form in the cytosol is not well understood. Recent studies suggest that zinc and the cytosolic thioredoxin system play a role in the biogenesis of these proteins. In the IMS, the mitochondrial import and assembly (MIA) pathway catalyzes both import into the IMS and oxidative folding of the small Tim proteins. Finally, assembly of the small Tim complexes is a multistep process driven by electrostatic and hydrophobic interactions; however, the chaperone function of the complex might require destabilization of these interactions to accommodate the substrate. Here, we review how folding of the small Tim proteins is regulated during their biogenesis, from maintenance of the unfolded precursors in the cytosol, to their import, oxidative folding, complex assembly and function in the IMS.

## 1. Introduction

Mitochondria are essential eukaryotic organelles harboring 1000–2000 different proteins. Approximately 99% of the total mitochondrial proteins are encoded by nuclear DNA, synthesized as precursors in the cytosol, and imported into mitochondria via elaborate transport machineries. The small Tim (translocases of the inner membrane) proteins are a group of small intermembrane space (IMS) proteins with a strictly conserved “twin CX_3_C” zinc-finger motif [1]. Members of the small Tim family are conserved and have been found in mammals, yeast and plants. *Saccharomyces cerevisiae*, for example, has five known small Tim proteins: Tim8, Tim9, Tim10, Tim12 and Tim13; of which Tim9, Tim10 and Tim12 are essential for cell viability [1–5]. The small Tim proteins play an essential chaperone-like role during the import of mitochondrial membrane proteins. They are in charge of chaperoning these hydrophobic membrane proteins through the aqueous mitochondrial IMS, transporting them from the translocase of the outer membrane (TOM) complex towards either the inner membrane (IM) or the outer membrane (OM) of mitochondria [6]. Their importance is illustrated by the fact that defects in human Tim8 lead to deafness dystonia syndrome [7,8]. For their function, the small Tim proteins assemble into at least two hexameric complexes, Tim9–Tim10 and Tim8–Tim13, where each complex includes three molecules of each subunit bound in an alternating form [9,10].

All mitochondrial IMS proteins are synthesized in the cytosol and have to be imported into mitochondria for their function. A unique feature for most of the IMS proteins is that their post-translational import is regulated by their cysteine redox state [11,12]. The biogenesis of the small Tim proteins is tightly coupled with their oxidative protein folding, which can be divided into four sequential steps: (i) In the cytosol the precursor proteins are kept in a reduced and unfolded form by cytosolic factors [13,14]; (ii) mitochondrial import of the reduced precursor proteins through the TOM complex [15]; (iii) oxidative protein folding in the IMS regulated by the mitochondrial import and assembly (MIA) pathway [16–18]; (iv) assembly of the oxidized, partially folded proteins into hexameric small Tim complexes [19,20]. All of these steps depend on the redox state of the conserved cysteine residues—while only reduced unfolded proteins can be imported into mitochondria, protein folding and complex formation requires disulfide bond formation. Here, we review the current knowledge regarding each step of the biogenesis and folding of the mitochondrial small Tim proteins with focus on Tim9 and Tim10. We will also discuss how the small Tim hexameric complex is assembled, and the mechanism by which this complex might perform its chaperone function.

## 2. Keeping the Precursor Protein Reduced and Unfolded in the Cytosol

**Figure 1:**
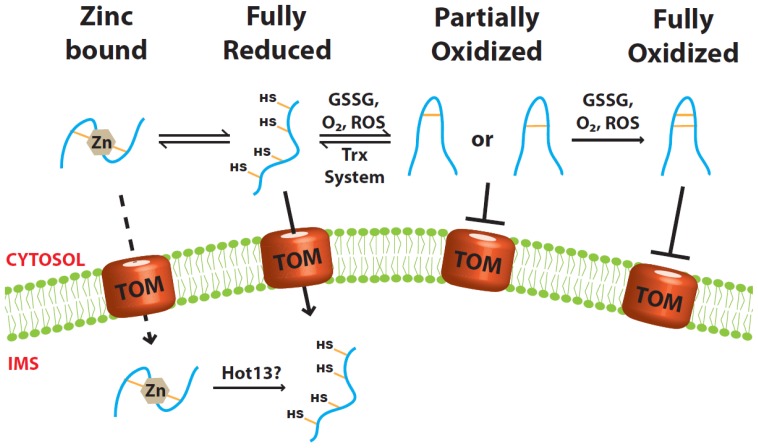
Only the Cys-reduced small Tim precursor proteins are import-competent. The small Tim proteins have to be kept in their reduced conformation while in the cytosol. Neither the fully nor the partially oxidized proteins can be imported into mitochondria. Oxidation could be brought about by GSSG, oxygen or reactive oxygen species (ROS). Partially oxidized proteins can be reduced by the cytosolic Thioredoxin system. Additionally, the reduced proteins could be stabilized by zinc binding, although it is uncertain whether the zinc-bound form can be imported directly.

Tim9 and Tim10 are two of the most important and best-characterized small Tim proteins. Circular dichroism and thiol-modification analyses showed that in the absence of a reducing agent the proteins are folded in an α-helical conformation stabilized by two pairs of intramolecular disulfide bonds [19,21,22]. The proteins become unfolded upon addition of a disulfide reducing agent (e.g., DTT, TCEP) even in the absence of a protein denaturant. Mitochondrial import analyses showed that the Cys-reduced states can be imported into mitochondria, whereas when oxidized the proteins are folded and import-incompetent [15,19,22] (Figure 1).

Glutathione, present in its reduced (GSH) and oxidized (GSSG) forms, is considered to be the major thiol-disulfide redox buffer of the cell. The total glutathione concentration in the cell is typically between 5 and 10 mM, and is mainly found in the reduced form in the cytosol and mitochondria [23]. *In vitro* studies have shown that GSSG at low micromolar concentrations is sufficient to oxidize the small Tim proteins and hinder their mitochondrial import [19,22]. Furthermore, once oxidized even 10 mM GSH is not sufficient to reduce the proteins [21,22]. The standard redox potentials for several small Tim proteins were determined [21,22] and showed similar values in the range of −0.31 to −0.33 V. Such low redox potentials are consistent with the oxidized proteins being stable in the mitochondrial IMS. However, because the IMS has a similar reducing environment to that of the cytosol [24], it suggests that precursor proteins could potentially be oxidized before they are imported. A few MIA pathway substrate proteins were recently shown to remain in the cytosol for several minutes before their import [25], which further reinforces the danger for precursor protein oxidation while in the cytosol. Therefore, after the precursor proteins are synthesized, a mechanism must exist to maintain them in a Cys-reduced and import-competent conformation, as well as to protect them from oxidative folding, aggregation and subsequent degradation in the cytosol. A recent study suggested that degradation of non-imported IMS precursor proteins is accomplished by the ubiquitin proteasome [26].

Chaperones are important players in the biogenesis of mitochondrial proteins [27–30]. In mammalian cells, Hsp70 and a mitochondrial import stimulation factor (MSF) were shown to target presequence-containing matrix precursors to mitochondria [30]. Furthermore, the Hsp70 and Hsp90 chaperones were identified to target IM carrier precursor proteins to mitochondria. In yeast, Hsp70 was shown to facilitate the import of both matrix and IM proteins [27–30]. However, so far no studies have been reported as to whether the cytosolic chaperones are involved in the import of mitochondrial IMS proteins. In the absence of evidence for the role of chaperones, two models have been proposed to keep the small Tim proteins in their Cys-reduced, import-competent conformation in the cytosol: zinc binding and the cytosolic thioredoxin (Trx) system.

### 2.1. Role of Zinc Ions

All the small Tim proteins contain a strictly conserved “twin CX3C” zinc-finger motif. *In vitro* experiments have shown that both Tim9 and Tim10 can bind Zn^2+^ in their reduced form, at a molar ratio of 1:1, with *K*_d_ values between 8.0 × 10^−8^ and 8.0 × 10^−10^ M^−1^ determined using different methods [2,31,32]. Upon binding, the proteins undergo a small yet detectable conformational change that can be seen by both far UV circular dichroism and fluorescence. Removal of any of the cysteines in Tim9 or Tim10 abolishes zinc binding, indicating that all four cysteines are involved in zinc coordination [13,21,32]. In the presence of zinc ions the rate of oxidative folding of Tim9 by GSSG is about 14-fold slower than that of the apo-proteins (no zinc). Thus, zinc-binding can stabilize the reduced form and keep it from oxidation [21,22]. However, though the total cellular zinc concentration is estimated to be somewhere between 0.1 and 0.5 mM [33], the free zinc concentration in the cells is believed to be very low (~10^−12^ M) [34–37]. Furthermore, experimental results showed that zinc is a strong inhibitor of the sulfhydryl oxidase Erv1, a component of the MIA pathway used for the import of the small Tim proteins. Consequently, at the zinc concentration that most of the small Tim proteins can bind zinc, their mitochondrial import is inhibited [13]. Therefore, if zinc does work as a chaperone in the cytosol, it may work in cooperation with another unknown factor and must be removed at some point during protein import. To this end, the IMS protein Hot13 (Helper of Tim) has been suggested to act as a zinc-chelator protein that is involved in assembly or recycling of the small Tim proteins, and helps maintain the MIA components in a zinc-free state [38,39].

### 2.2. Role of the Cytosolic Redoxin Systems

A second model is based on our recent finding that the cytosolic Trx system facilitates the import of mitochondrial small Tim proteins [14]. Thioredoxins and glutaredoxins (Grx) are ubiquitous oxidoreductases in charge of thiol regulation and oxidative stress defense. Their main function is to reduce disulfide bonds found in proteins [40]. In the yeast *S. cerevisiae*, the Trx system comprises Trx1, Trx2, Trx reductase and NADPH. Trx1 and Trx2 act on substrate proteins while Trx reductase takes electrons from NADPH and transfers them to Trx1 and Trx2. Durigon *et al*. [14] recently showed that a Δ*trx1trx2* double deletion mutant has a strong growth defect under respiratory conditions. The mutant strain also has lower mitochondrial levels of the small Tim proteins. Importantly, mitochondrial import studies confirmed that the import levels of both Tim9 and Tim10 increased significantly in the presence of the Trx1 system. A similar result was also obtained for Cox19, a CX_9_C motif-containing substrate of the MIA pathway. Furthermore, an efficient disulfide bond transfer reaction was reconstituted using purified Tim10 and the Trx1 system. *In vitro* assays have also shown that Trx1 is capable of reducing the partially oxidized, one-disulfide-bonded intermediates of Tim10, while the fully oxidized protein was Trx-resistant [14]. These results suggest that the Trx system counterbalances the oxidative folding in the cytosol by reducing any early folding intermediates, which were shown to be import-incompetent as well [22].

A very recent study based on *in vivo* NMR analysis showed that overexpressed human Mia40 (a twin CX_9_C motif-containing protein) was oxidatively folded in the cytosol of human embryonic kidney cells, and its folding state depended on the cytosolic Grx1 system and to a lesser extent on the Trx1 system [41]. Interestingly, while overexpressed human Mia40 is largely trapped in the cytosol, it has been shown that overexpressed yeast Mia40 is successfully imported into yeast mitochondria [42]. This difference may be because human Mia40 does not have an *N*-terminal mitochondria-targeting signal and is imported through the MIA pathway, while yeast Mia40 does have an *N*-terminal targeting signal and is imported via the translocase of the inner membrane 23 (TIM23) pathway [43–46]. However, purified human Grx1 cannot reduce human Mia40 *in vitro*, thereby implying an indirect link to the redox state of Mia40 [41].

In summary, both zinc-binding and the cytosolic Trx system can maintain the small Tim proteins in Cys-reduced forms. Due to its inhibitory activity, zinc alone is unlikely to be the major player in maintaining the precursors in their import-competent form in the cytosol. The Trx system not only facilitates the import of the small Tim proteins, but also of non-zinc binding proteins such as Cox19. Indeed, both the Trx system and the human Grx system are able to keep the CX_9_C motif precursors proteins in their reduced form by preventing their oxidative folding at an early stage, and can thus facilitate the import of mitochondrial IMS proteins. Therefore, these systems appear to be an important primary factor in mediating the biogenesis of the redox-sensitive IMS proteins by reductively unfolding the proteins. Whether the cytosolic Grx system, particularly the yeast mitochondrial OM-anchored Grx2 [47], also plays a role in the import of the small Tim proteins is unknown. More studies are still required to understand the detailed molecular mechanisms of protein folding/unfolding in the cytosol.

## 3. Import, Folding and Oxidation

The small Tim proteins are found in their oxidized states in purified mitochondria and *in vivo* [19]. The identification of a disulfide relay system (Mia40-Erv1 system) in the mitochondrial IMS indicated that oxidative protein folding occurs in the IMS and completely changed the view of how IMS proteins fold and get imported [11,12]. For a long time it was believed that the IMS, like the cytosol, has a reduced environment and thus would not support protein disulfide bond formation. Conversely, oxidative protein folding has now been suggested as the reason many IMS proteins, including the small Tim proteins, are trapped inside the IMS [15,16]. In contrast to the less studied and ill-defined mechanism in the cytosol, many studies have been carried out in understanding the Mia40-Erv1 system (MIA pathway) and the molecular mechanism of oxidative protein folding in the mitochondrial IMS. There are many excellent reviews about this disulfide relay system [11,12,48,49] and it will, therefore, only be explained briefly in this review. We will instead focus on the import and folding of the small Tim proteins, which are proven substrates of the MIA pathway.

### 3.1. Disulfide Relay System of the IMS

The MIA machinery localized inside the mitochondrial IMS was found to be required for import of many cysteine-rich IMS proteins, including the small Tim proteins [44,45,50]. The system includes two essential proteins: the disulfide carrier Mia40 that recognizes and transfers disulfides to newly imported substrate proteins, and the sulfhydryl oxidase Erv1 that reoxidizes reduced Mia40. Lastly, both oxygen and cytochrome *c* can act as the electron acceptors of Erv1 [11].

### 3.2. Import of the Small Tim Proteins

**Figure 2:**
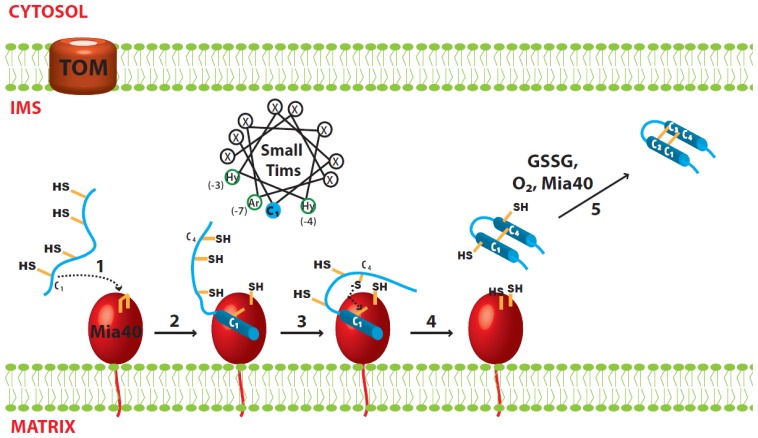
Proposed model for oxidative folding of the small Tim proteins. (1) Nucleophilic attack of the C_1_ cysteine from the small Tim proteins; (2) Mia40-dependent folding initiated by hydrophobic interactions at the MISS/ITS segment, and stabilized by formation of an intermolecular disulfide bond; (3) Nucleophilic attack of the C_4_ cysteine from the small Tim proteins; (4) Mia40-independent folding. The second helix is formed using the first helix as scaffold, and stabilized by formation of the first intramolecular disulfide bond in the small Tim proteins (C_1_–C_4_); (5) The second disulfide bond could be formed by either oxygen, GSSG or Mia40.

The small Tim proteins, like other mitochondrial proteins, reach the IMS by passing through the TOM complex in the OM. All small Tim proteins have an internal targeting signal called MISS (mitochondrial IMS sorting signal), or ITS (IMS-targeting signal). Inside the IMS, Mia40 functions both as an import receptor and a disulfide carrier. The MISS/ITS includes a cysteine residue that is important for mitochondrial import and docking to Mia40 (as a receptor) [51,52]. Systematic mutagenesis studies showed that, for Tim9 and Tim10, the MISS/ITS consists of 9 amino acids upstream of the first (*N*-terminal) cysteine (C_1_). Apart from the docking cysteine, hydrophobic residues at positions −3, −4, as well as an aromatic residue at position −7 from the cysteine are important; the consensus sequence is thus X[Ar]XX[Hy][Hy]XXC (Ar: aromatic residue; Hy: hydrophobic residue). These residues are arranged such that they point towards the same side of the α-helix (Figure 2). Indeed, an upstream or downstream shift of the docking C_1_ cysteine in Tim9, where upon the distance from the hydrophobic residues to the cysteine is changed by a full helix turn, strongly impairs binding of the protein to Mia40 and its mitochondrial import [52]. This is because the hydrophobic residues are no longer in contact with the cleft in Mia40. On the other hand, swapping the MISS/ITS from the *N*-terminal to the *C*-terminal end of Tim10 does not affect its import, indicating its mitochondrial import occurs post-translationally. Furthermore, the MISS/ITS peptide is also sufficient for targeting non-mitochondrial proteins to the mitochondrial IMS [51,52].

### 3.3. Oxidative Folding of the Small Tim Proteins

Upon interaction with Mia40, the MISS/ITS becomes folded and forms an amphipathic helix that has the crucial hydrophobic and aromatic residues on one side and the non-essential residues on the other side. The hydrophobic side of the helix fits well into the hydrophobic cleft of Mia40. The folding of the small Tim MISS/ITS is induced by Mia40 via these hydrophobic interactions, and is further stabilized by formation of an intermolecular disulfide bond between the docking cysteine (C_1_) of small Tims and a redox active cysteine of Mia40 [16–18,53]. Thus, these hydrophobic residues are crucial for the initial folding as they are precisely positioned to interact with Mia40 [18,51,52]. However, how the small Tim proteins become fully folded and oxidized after their initial interaction with Mia40 is still unclear.

The sequence of events after substrate recognition by Mia40 is better understood when looking at the folding steps of another MIA pathway substrate, human Cox17 (hCox17). hCox17 is part of a group of IMS proteins with a conserved twin CX_9_C motif. A study by Banci *et al*. [18] showed that oxidation and folding are coordinated events in the hCox17 protein. The MISS/ITS segment of the protein is downstream of the docking cysteine Cys45 (C_3_) and initially binds the hydrophobic cleft of Mia40, forming an intermolecular disulfide bond. Upon binding, the MISS/ITS segment folds into an α-helix spanning 15 residues downstream of the docking cysteine, while the rest of the protein remains unfolded [18]. In the absence of the docking cysteine the protein can still bind Mia40, albeit with a weaker affinity. Moreover, without the formation of the intermolecular disulfide bond the MISS/ITS segment remains unfolded [18]. Therefore, a disulfide bond is required to stabilize the initial folding of hCox17. The second step involves the nucleophilic attack of Cys36 (C_2_) to break the intermolecular disulfide bond. The reaction creates the internal C_2_–C_3_ disulfide in hCox17 while, simultaneously, the second α-helix is formed. Interestingly this step is independent of Mia40-hCox17 uses the hydrophobic side of its first helix as a scaffold for the formation of the second helix [18]. Here, again, disulfide bond formation (oxidation) is coordinated with protein folding. After these two folding steps hCox17 has acquired the same secondary structure as the fully mature protein, but is still lacking one disulfide bond. This agrees with another finding showing that the outer disulfide bond (C_1_–C_4_) of yeast Cox19 (another CX_9_C protein) cannot be formed in the absence of the inner disulfide (C_2_–C_3_) [54]. The outer disulfide bond can be formed by different components depending on the surrounding conditions. Under aerobic conditions oxygen can easily oxidize the cysteines to form the second disulfide on an already-folded protein [55], whereas Mia40 can also completely oxidize the protein when found in vast excess [54]. Alternatively, *in vivo* assays have also suggested that Erv1 forms a tertiary complex with Mia40 and the small Tim proteins under certain conditions [56], thus creating the opportunity for Erv1 to insert the second disulfide.

The folding of small Tim proteins is less understood, mainly because the reduced proteins at high concentration tend to degrade and render any NMR analysis difficult [18]. Unlike hCox17, the small Tim proteins have a twin CX_3_C motif, their docking cysteine is C_1_ and the MISS/ITS segment is upstream of C_1_. This means the outer disulfide is likely formed before the inner disulfide, contrary to what is seen for hCox17. Indeed, a double mutation of the outer disulfide cysteines (C1,4S) strongly inhibits the Tim9 interaction with Mia40 [16]. Additionally, a peptide corresponding to the MISS/ITS segment of Tim9 including the C_1_ cysteine (RLYSNLVERC) was shown to bind Mia40 through an intermolecular disulfide bond [18,52]. Upon binding Mia40, the previously unfolded peptide was able to adopt a stable α-helix conformation [18]. Thus, the Mia40-induced folding of the MISS/ITS segment appears to be general for proteins with either a twin CX_3_C or CX_9_C motif (Figure 2). How the second disulfide bond is formed is an important controversial question and more experiments are needed to address it.

### 3.4. Oxidized Tim9 and Tim10

The fully oxidized small Tim proteins have a helix-loop-helix structure where the two helices are linked and stabilized by two intramolecular disulfide bonds, C_1_–C_4_ and C_2_–C_3_ [9,10,57]. The disulfide bonds are required to maintain the secondary structure of the proteins and also render them more resistant to trypsin digestion [19,31]. Sedimentation equilibrium studies showed that in a condition mimicking the physiological environment (Tris-HCl, pH 7.4, 150 mM NaCl) Tim9 forms a dimer and Tim10 a monomer [20,58]. No information is known regarding the oligomerization state of other small Tim proteins. So far no crystal structure of the individual small Tim proteins has been published. However, NMR studies have shown that both the N- and C-terminus of Tim10 are unstructured, whereas only the C-terminus of Tim9 is flexible [31]. Overall, both Tim9 and Tim10 exist in a molten-globule state in which secondary structures are formed, but the proteins do not pack together in a unique way [31]. Far UV circular dichroism studies showed that the individual oxidized small Tim proteins have similar secondary structure to that of the proteins in the hexameric complex [31].

## 4. Complex Assembly

### 4.1. Assembly Process of the Tim9–Tim10 Complex

**Figure 3:**
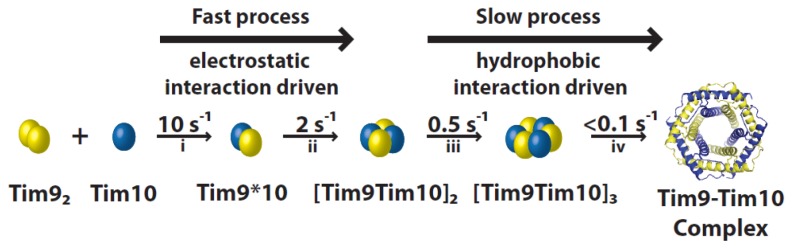
Tim9–Tim10 hexameric complex assembly. The process can be separated into four kinetic steps: formation of a heterodimer (i), a tetramer (ii), a hexamer (iii) and a final hydrophobic rearrangement (iv). The two initial fast steps are controlled mainly by electrostatic interactions, whereas the final two slow steps are driven by hydrophobic interactions.

Formation of the hexameric Tim9–Tim10 complex from the individual subunits can be divided into four kinetic steps (Figure 3). Stopped-flow experiments coupled with mutagenesis showed clear salt concentration dependence in the rate of assembly in the first two steps, but not in the last two steps. Furthermore, the overall rate of assembly depends on the pH in a bell-shaped profile, with two pKa values similar to the isoelectric points of Tim9 and Tim10.

The initial step consists in the formation of a Tim9*10 heterodimer. Monomeric Tim10 binds allosterically to dimer Tim9, substituting one of the Tim9 subunits [20]. Two factors could favor heterodimerisation: (i) the central loop structures of Tim9 and Tim10 complement each other better than if they were to form an homodimer; (ii) Aryl groups in Tim9 and methionine residues in Tim10 interlace in the intersubunit surface, and this arrangement appears to improve the fit of the heterodimer [10]. These interactions with Tim9 also cause the flexible Tim10 structure to become more defined [31]. Once formed, the dimers quickly associate to form a tetramer further stabilized by electrostatic interactions. This is consistent with the presence of important intersubunit salt bridges formed between both sides of the Tim9/10 interfaces [59]. The third step includes the slow incorporation of another Tim9*10 dimer to form a hexameric complex. Lastly, hydrophobic-driven rearrangements produce the final complex [20,60]. As this multistep process occurs efficiently *in vitro*, there is not thought to be any cofactor assisting it. Interestingly, the major hydrophobic areas located between the *N*- and *C*-terminal helices may be less protected in the later hexameric assembly intermediates, suggesting that these complexes could play a role in substrate binding.

### 4.2. Structure of the Tim9–Tim10 Complex

**Figure 4:**
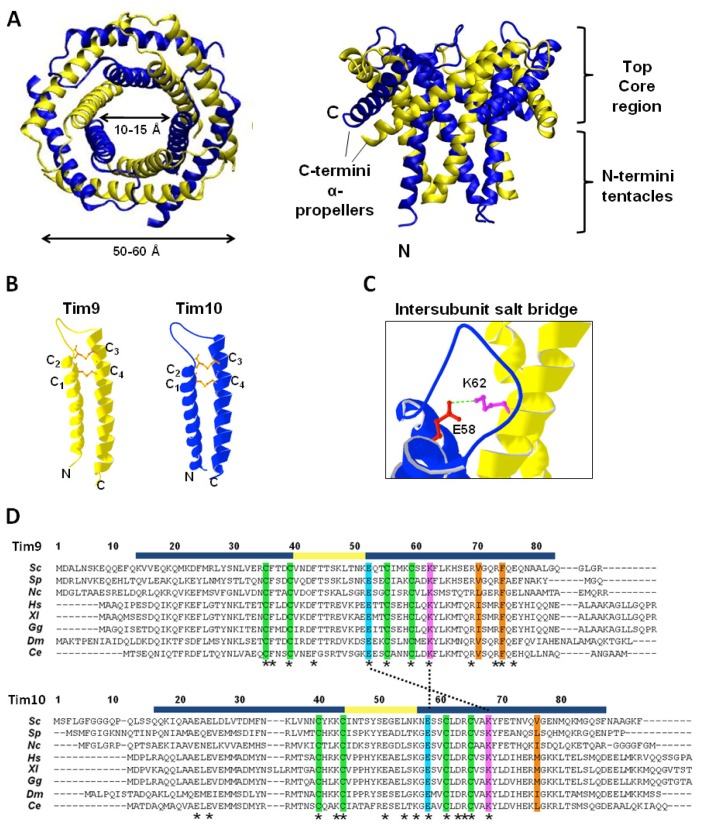
Tim9–Tim10 hexameric complex. (**A**) Crystal structure of yeast Tim9–Tim10 complex, top (**left**) and side (**right**) view respectively (PDB: 3DXR). Tim9 (yellow) and Tim10 (blue). The outer layer is formed by the six *C*-terminal helices (α-propellers) and the inner layer by the six *N*-terminal helices (tentacles); (**B**) Structure of Tim9 and Tim10 showing the two helices linked by intramolecular disulfide bonds (orange) (PDB: 3DXR); (**C**) Conserved salt bridge between a glutamate residue in the loop of Tim10 and a lysine residue in the outer helix of Tim9; (**D**) Alignment of Tim9 and Tim10 amino acid sequences. Fully conserved residues are marked with asterisks (*). Helical regions and the central loop are marked in blue and yellow respectively. Conserved cysteine residues are highlighted in green. Key charged residues are highlighted in pink (positively charged) and light blue (negatively charged), and salt bridges between them are marked with a dotted line. Central residues of hydrophobic clusters are highlighted in orange. *Sc: Saccharomyces cerevisiae*, *Sp: Schizosaccharomyces pombe*, *Nc: Neurospora crassa*, *Hs: Homo sapiens*, *Xl: Xenopus laevis*, *Gg: Gallus gallus*, *Dm: Drosophila melanogaster*, *Ce: Caenorhabditis elegans*.

Oxidative folding in the IMS allows the arrangement of the small Tim proteins into hexameric complexes. The crystal structures of the human and yeast Tim9–Tim10 complex have been solved, as well as that of the yeast Tim8–Tim13 complex [9,10,59]. All three complex structures are similar, adopting a donut-shaped configuration with an outer diameter of 50–60 Å, and an inner pore of 10–15 Å (Figure 4A). The structures resemble those of the prefoldin and Skp chaperones in terms of overall architecture [61–63]. The Tim9–Tim10 hexamer consists of three molecules of each subunit, arranged in a circle, with an inner, downward-pointing circle made from the subunits N-termini (often referred to as “tentacles” as in prefoldin and Skp), and an outer, outward-pointing circle (“α-propellers”) made from the *C*-termini tilted outwards at 60° [10]. The steric bulk of the disulfide bonds in the individual subunits generate the characteristic structure of the complex, by causing the helices to splay outwards [10,59] (Figure 4B). Intermolecular salt-bridges between lysine and glutamate residues are critical to complex stability, while hydrophobic residues in the core are sequestered by junctions formed between inner and outer helices with the intermolecular contacts generally occurring in the *N*-terminal regions of the inner helices, and in the core regions of the outer helices (Figure 4C,D). Additionally, hydrophobic residues form clusters in which two residues from one subunit (e.g., Tim9 V70 and F74) are surrounded by several hydrophobic residues from the neighboring subunit [64]. There is not an obvious hydrophobic pocket for substrate binding, suggesting that a conformational change is required for the chaperone function of the Tim9–Tim10 complex.

### 4.3. Stability of the Tim9–Tim10 Complex

Various parts of Tim9 and Tim10 have been studied for their role in complex formation and stability using purified proteins, *in organelle* analysis, and *in vivo* approaches. The importance of the N- and C-terminus of Tim9 and Tim10 in complex stability has been studied both *in vitro* and *in vivo*. Deletion of the C-terminus of Tim10 inhibits its interaction with Tim9 and, therefore, stops complex assembly. Similarly, a deletion of the C-terminus of Tim9 destabilized the complex. In contrast, mutation of the N-terminus of either Tim10 or Tim9 showed no considerable effect on complex formation [65]. Together, these data suggest that the C-termini (“propeller blades”) of Tim9 and Tim10 are significantly more important for hexameric complex formation/stability than the N-termini (“tentacles”).

The roles of specific amino acids of Tim9 and Tim10 in complex stability have also been investigated. The four conserved cysteine residues in the proteins form two pairs of intramolecular disulfide bonds while in the complex. They are required for complex formation and constricting the neck of the fixed-angle helical hairpin [59]. Their importance is highlighted by the human Tim8 cysteine mutation, C66W, found to cause deafness dystonia syndrome [7,8]. *In vitro* studies showed that reduced proteins in the presence or absence of zinc, or after blocking of the cysteines, are prevented from forming the hexameric complex [7,19,31,66]. Incorporation into the complex shields the disulfide bonds, making them resistant to DTT [19], whereas the individual subunits can be reduced [1,22]. A recent study showed that loss of any disulfide bond results in greatly reduced complex formation *in vivo* and instability of both proteins, but surprisingly has little effect on cell growth [67].

Other important stabilizing interactions are the intersubunit salt-bridges buried at the Tim9 and Tim10 interface [59]. Particularly important to these interactions are the glutamate residues Tim9E52 and Tim10E58 (*N*-terminal to the third cysteine in both Tim9 and Tim 10), and the nearby lysine residues Tim9K51 and Tim10K56 [59,68]. Stability of the Tim9–Tim10 complex in mitochondria is heavily disrupted by mutation of any of these residues [42,46,47]. These charged residues are highly conserved and located in the core region of the complex. The key intersubunit interactions are formed by the Tim9E52-Tim10K68 and the Tim9K62-Tim10E58 salt-bridges. However, as with the conserved cysteine residues, mutation of these charged residues, while significantly affecting detection of the Tim9–Tim10 complex, did not necessarily result in cell death. For example, the *tim9–19* mutant (a Tim9E52G mutation) does not form detectable levels of Tim9–10 complex, but still supports slow cell growth [69].

In contrast to the role of the cysteines and the salt-bridge interactions, the hydrophobic interactions between adjacent subunits are potentially more dynamic [59,64]. *In vitro* experimental studies and computational simulations suggested that dynamics of the hydrophobic interactions play a subtle and yet important role in stabilizing and regulating the function of the Tim9–Tim10 complex. Two hydrophobic clusters (A and B) were identified, each centered on key residues (Figure 3D, Val70 and Phe74 of Tim9 for cluster A, Val76 of Tim10 for cluster B) from one subunit that are surrounded by several hydrophobic residues from the other subunit [64]. Interestingly, at increased temperatures the stabilizing effect of these hydrophobic interactions is predicted to become destabilizing, which is in agreement with experimental results of a decreased rate of late-stage complex assembly at elevated temperatures [64]. However, more experiments are required to know whether mutations of these hydrophobic residues disrupt complex formation or simply disturb its function in import of its substrate proteins.

Tim9 and Tim10 have also been shown to form a ternary hexameric complex with Tim12. Tim12 is an essential IMS protein in yeast associated peripherally with the translocase of the inner membrane 22 (TIM22) complex of the IM [70]. Tim12 is believed to link the Tim9–Tim10 complex with the TIM22 translocase during import of Tim9–Tim10 substrates. Although the Tim9–Tim10–Tim12 complex is less studied, it appears to consist of three Tim9, one Tim10, and two Tim12 [71]. Whether the complex forms through displacement of Tim10 molecules by Tim12 or through *de novo* interaction with free Tim9 and Tim10 subunits is not currently known.

## 5. Functional Mechanism of the Small Tim Proteins

The biological function of the small Tim proteins is to assist in the import of hydrophobic proteins into the mitochondrial inner and outer membranes [2–4,6,72]. The exact mechanism of substrate interaction and release from the small Tim proteins is not yet known. It is also not clear which form of the proteins, Tim9, Tim10 or the Tim9–Tim10 complex acts as the functional chaperone during the import of their substrate proteins; while it is generally believed that the hexameric complexes are essential for the chaperone function, some studies suggest that complex formation might not necessarily be required. Moreover, there is some level of substrate specificity between the two homologous small Tim complexes: the Tim8–Tim13 complex has been linked to Tim23 insertion [73], while Tim9–Tim10 is involved in the insertion of a wide range of OM and IM proteins, in particular the carrier proteins of the IM [2,3]. Here, we summarize the experimental results and proposed models that are based on the most studied model substrate of Tim9–Tim10: the ATP/ADP Carrier (AAC) protein.

### 5.1. Import of AAC

**Figure 5:**
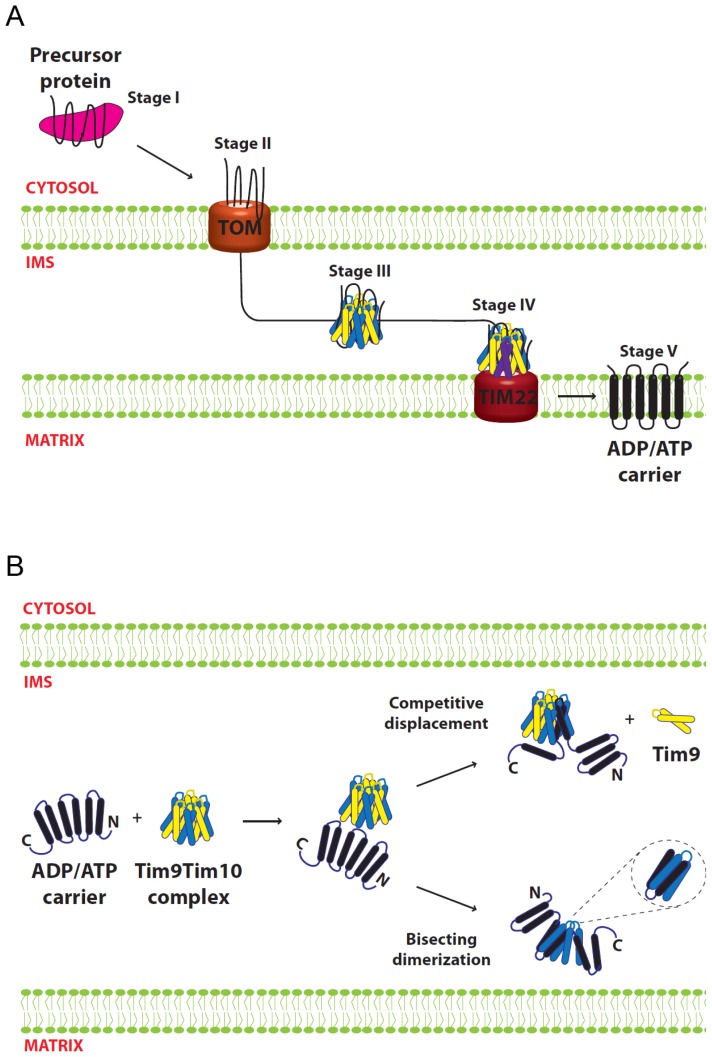
Small Tim function in carrier protein biogenesis. (**A**) Biogenesis of AAC. Stage I, precursor in the cytosol is bound by chaperones. Stage II, precursor engages the TOM complex at the mitochondrial OM. Stage III, precursor is bound by the small Tim proteins and chaperoned through the IMS. Stage IV, precursor interacts with Tim12 (in purple) and reaches the TIM22 complex. Stage V, the carrier protein is fully inserted in the IM and dimerizes into its functional form; (**B**) Model for small Tim proteins interaction with the AAC substrate. AAC binding is initiated through the N-termini of the small Tim subunits. AAC could either substitute a Tim9 subunit (competitive displacement) or cut across some of the complex subunits (bisecting dimerization). The two models are not mutually exclusive and at least one Tim9 subunit remains in close proximity to the substrate.

AAC, a mitochondrial IM protein, is one of the best-studied proteins in terms of its import and interaction with the small Tim proteins [2–4]. It belongs to a large family of IM carrier proteins that contain non-cleavable targeting sequences. AAC spans the IM six times comprised of three modular repeats of paired transmembrane domains. In contrast to the small Tims and many other mitochondrial proteins, AAC and carrier proteins are not necessarily imported into mitochondria as a linear polypeptide chain, and proper import appears to be dependent on the full intact protein [74,75]. Import of AAC can be divided into five stages [76–78]. The initial interaction with cytosolic chaperones (stage 1) is followed by association with the TOM complex (stage 2) in an ATP-dependent manner. Then the precursor passes through the IMS chaperoned by the small Tim proteins (Stage 3), followed by a membrane potential-dependent IM insertion by the TIM22 complex (Stage 4). Finally, the AAC is released and assembled into its functional form in the IM (Stage 5) (Figure 5A).

### 5.2. Models for Tim9–Tim10 Chaperone Function

The chaperone function of the small Tim proteins can be conceived as a multi-step process. It requires a mechanism that can both recognize and bind the substrate, and then release it. The first model for small Tim function proposed that the substrate binding is provided by individual Tim10 molecules, with the role of Tim9 being to stabilize Tim10 when it is not interacting with substrate. This model was proposed mainly based on studies showing that Tim10 can interact with AAC, whereas no stable interactions between Tim9 and AAC were detected using the same approaches (NMR, protein cross-linking and peptide spot assay) [2,4,10,75]. Furthermore, the Tim10-AAC interactions were severely disrupted by deletion of 30 amino acids at the Tim10 *N*-terminus [65], indicating a critical role for the *N*-terminal of Tim10 in substrate interaction. It was suggested that the loop regions of Tim10 line up in anti-parallel with AAC matrix loops (“carrier signatures”), with hydrophobic and complementary charged residues making contact [2]. However, Tim9 does not contain these complementary sequences and would therefore be unable to interact with AAC [4]. Instead, Tim9 function might be explained by the observation that mutations of *tim9* that result in low protein levels correlate with a reduction in Tim10 protein [4,67], suggesting that Tim10 is only stable in the presence of Tim9. As such, the interaction with Tim9 serves only to protect Tim10 from Yme1-mediated degradation. A simple model generated from these data is that Tim10 interacts and chaperones precursor AAC, while Tim9 acts to maintain an available pool of Tim10 in the absence of substrate.

The crystal structure of the mammalian Tim9–Tim10 complex supported the idea that Tim10 is the primary protein involved in substrate binding [10]. In contrast to the homologous Tim8–Tim13 complex, which can be modeled to encompass its relatively small substrate Tim23 in its central cavity [9], the structure of the Tim9–Tim10 hexameric complex does not appear to accommodate the AAC molecule, and the hydrophobic residues predicted to interact with the substrate are buried in intersubunit interactions [8,42]. Thus, substrate binding most likely takes place through conformational changes and/or subunit rearrangements that expose the binding surfaces of Tim10. Two models were proposed for how the substrate-binding regions might transition from being buried in the Tim9–Tim10 hexamer to binding AAC. In each case, one subunit is displaced and replaced by two helices of AAC. In the bisecting dimerization model, two transmembrane helices of AAC cut across the tentacles of a subunit in a tail-to-tail orientation. In the competitive displacement model, transmembrane helices four and five (H4, H5) of AAC displace a Tim9 subunit, with the H4–H5 loop of AAC positioned similarly to the loop regions of the small Tim proteins (Figure 5B). This model could explain why the AAC H4–H5 segment showed strongest binding to Tim10 in peptide spot assays [79,80]. The two models are not mutually exclusive but explain available experimental results from different angles, and they could work in combination allowing the rest of the substrate to fit within the complex [8]. In support of these models, the presence of a low-populated excited state of Tim9–Tim10 complex with disrupted intersubunit hydrophobic interactions would allow the complex to “breathe”, thus providing an explanation for the initial substrate interaction with Tim10 [64]. In this scenario, some complexes will be more thermodynamically stable (“closed”) and some will be more energetic (“open”). In the open phase some hydrophobic residues are exposed allowing Tim10 to interact with incoming precursors.

As discussed in Section 4.3, no consistent picture appears connecting the status of the Tim9–Tim10 complex, its interaction with substrate and cell viability. Interestingly, some mutations lack detectable Tim9–Tim10 hexameric complex on blue-native gels, but still can import AAC [42,45,46,57], suggesting the complex is not required for AAC import. However, several lines of evidence support the involvement of both Tim9 and Tim10 in substrate interaction. For example, Tim9 can co-immuno-precipitate AAC [80], and crosslinks between AAC and Tim9 are detectable under certain conditions [3,81]. Introduction of a fifth cysteine residue into Tim9 allowed clear crosslinking of Tim9 to AAC [56,57], strongly suggesting that these proteins are in close proximity. Furthermore, a Tim9 *N*-terminal deletion mutant, Tim9ΔN10, in which Tim10 remains stable, does not properly import AAC *in vitro* [59]. Thus, overall it seems that both Tim9 and Tim10 are in proximity to the substrate, and the presence of both proteins is required for normal substrate interaction. While Tim10 might provide the majority of the binding surfaces for AAC, it is more useful to view both Tim9 and Tim10 acting in concert to chaperone substrates through the IMS.

Taken together, the available data supports a model (Figure 5B), in which Tim9–Tim10 complex recognizes incoming substrate through the *N*-terminal tentacle regions of Tim9 and Tim10. This recognition triggers opening of the complex by promoting destabilization of the hydrophobic interactions between subunits, enabling helices of AAC to be incorporated into the complex and displacing a Tim9 subunit in the process via the “competitive displacement” mechanism. It is likely that, while Tim10 provides most of the substrate-binding capacity, at least one Tim9 subunit remains in close proximity to AAC during its transit through the IMS. It must be stated, however, that the models proposed remain speculative. Structural information on the conformation of the small Tim proteins while engaged in binding to substrate is required to fully understand the functional mechanism of the small Tim chaperones.

## 6. Conclusions

The biogenesis and function of the mitochondrial small Tim proteins is a process requiring correct protein folding at both a spatial and temporal level. Much of this process is centered on the four strictly conserved cysteine residues in each protein. The cysteines must remain reduced for the proteins to be imported into mitochondria. As a result, oxidative folding regulates the import of individual proteins, their stability, and their final assembly into a functional complex. The function of the small Tim proteins in chaperoning mitochondrial membrane precursors might, on the other hand, depend on more complicated, dynamic hydrophobic interactions. Despite the knowledge gained thus far, much remains to be investigated. For example, how the redoxin systems, metal ions, cytosolic chaperones and the proteasome all interact in the maintenance and degradation of the small Tim proteins while in the cytosol. Additionally, the detailed oxidative folding pathway of the small Tim proteins in the IMS remains a speculative model, with the proposed steps and order of disulfide bond formation still requiring experimental backup. Lastly, deciphering how the small Tim complexes remodel to incorporate both different subunit compositions, as well as incoming substrates, should help clarify the functional molecular mechanism of these chaperone proteins. Answering these and other questions will provide a clearer picture of oxidative protein folding in the IMS and the essential roles the small Tim proteins play in mitochondrial protein biogenesis.
